# Extracellular Vesicles, the Road toward the Improvement of ART Outcomes

**DOI:** 10.3390/ani10112171

**Published:** 2020-11-21

**Authors:** Maria G. Gervasi, Ana J. Soler, Lauro González-Fernández, Marco G. Alves, Pedro F. Oliveira, David Martín-Hidalgo

**Affiliations:** 1 Department of Veterinary & Animal Sciences, University of Massachusetts, Amherst, MA 01003-9301, USA; mariagracia@vasci.umass.edu; 2 IREC (CSIC-UCLM-JCCM), ETSIAM, Campus Universitario, s/n, 02071 Albacete, Spain; anajosefa.soler@uclm.es; 3 Research Group of Intracellular Signalling and Technology of Reproduction (Research Institute INBIO G+C), University of Extremadura, 10003 Cáceres, Spain; lgonfer@unex.es; 4 Department of Biochemistry and Molecular Biology and Genetics, Faculty of Veterinary Sciences, University of Extremadura, 10003 Cáceres, Spain; 5 Unit for Multidisciplinary Research in Biomedicine (UMIB), Laboratory of Cell Biology, Department of Microscopy, Institute of Biomedical Sciences Abel Salazar (ICBAS), University of Porto, 4050-313 Porto, Portugal; alvesmarc@gmail.com; 6 Department of Chemistry, University of Aveiro, 3810-193 Aveiro, Portugal; p.foliveira@ua.pt; 7 Department of Physiology, Faculty of Veterinary Sciences, University of Extremadura, 10003 Cáceres, Spain

**Keywords:** spermatozoa, oocyte, in vitro fertilization, extracellular vesicles, assisted reproductive technologies, embryo

## Abstract

**Simple Summary:**

Nowadays, the farm and pet industries cannot be sustained without assisted reproductive technologies (ART). Nevertheless, ART outcomes still are far from ideal. Recently, the emerging role of bioactive molecules—known as “extracellular vesicles” (EVs)—in the reproductive processes has been reported. EVs originate in different sections of the reproductive tract, and they can be isolated from reproductive fluids. Here, we update recent advances in the use of EVs as additive to media used in ART to enhance their reproductive outcome, mainly in domestic mammal animals.

**Abstract:**

Nowadays, farm animal industries use assisted reproductive technologies (ART) as a tool to manage herds’ reproductive outcomes, for a fast dissemination of genetic improvement as well as to bypass subfertility issues. ART comprise at least one of the following procedures: collection and handling of oocytes, sperm, and embryos in in vitro conditions. Therefore, in these conditions, the interaction with the oviductal environment of gametes and early embryos during fertilization and the first stages of embryo development is lost. As a result, embryos obtained in in vitro fertilization (IVF) have less quality in comparison with those obtained in vivo, and have lower chances to implant and develop into viable offspring. In addition, media currently used for IVF are very similar to those empirically developed more than five decades ago. Recently, the importance of extracellular vesicles (EVs) in the fertility process has flourished. EVs are recognized as effective intercellular vehicles for communication as they deliver their cargo of proteins, lipids, and genetic material. Thus, during their transit through the female reproductive tract both gametes, oocyte and spermatozoa (that previously encountered EVs produced by male reproductive tract) interact with EVs produced by the female reproductive tract, passing them important information that contributes to a successful fertilization and embryo development. This fact highlights that the reproductive tract EVs cargo has an important role in reproductive events, which is missing in current ART media. This review aims to recapitulate recent advances in EVs functions on the fertilization process, highlighting the latest proposals with an applied approach to enhance ART outcome through EV utilization as an additive to the media of current ART procedures.

## 1. Assisted Reproductive Technologies and Their Handicaps

Countless advantages can be quoted for the use of assisted reproductive technologies (ART). ART have been used to preserve valuable genetic material (cryobiology), to perform offspring sex selection (by sperm sorting), to reduce the incidence of venereal diseases (by artificial insemination (AI)), to bypass sub-fertility issues (by in vitro fertilization (IVF) or intracytoplasmic sperm injection (ICSI)), to increase reproductive outcomes and maximize the number of offspring that can be obtained by a single female (by inducing superovulation, performing IVF and eventually transferring embryos to female recipients by embryo transference (ET)), and to enhance reproduction of males (by increasing the performance of a single ejaculate that can be cryopreserved and then used by AI). Consequently, ART have a critical role on the management of the herd; for example, a high proportion of pigs and bovines are produced by AI [1,2].

One of the most popular ART used is IVF. Current IVF protocols are based on the basic knowledge provided in 1951 by two investigators that independently discovered that ejaculated mammalian sperm require a period of incubation in the female reproductive tract to acquire the ability to fertilize [3,4]. This phenomenon is described as sperm capacitation [5], and it settled the cornerstone for the development of IVF [6]. Twenty years after the description of sperm capacitation, the first successful IVF in mouse using a defined medium in absence of female fluids was performed [7]. Before that, successful IVF were performed by including female fluids from the reproductive tract in the incubation media [8,9]. These results highlighted the importance of factors present in female reproductive fluids to accomplish fertilization. Nevertheless, current IVF media do not differ than those developed empirically more than 50 years ago [7].

It is well documented that the quantity and quality of embryos obtained by ART are lower in comparison to those obtained in vivo by mating [10,11]. In addition, ART-derived embryos present lower chances to fully develop and derive in live offspring [10,11], underlining the need to enhance the current media used during ART protocols. It is clear that current ART procedures lack the interaction of gametes with several components present in the reproductive tract during fertilization and the first stages of development. Recent advances point out that extracellular vesicles (EVs) present in the reproductive environment help to achieve in vitro-derived embryos with development levels similar to in vivo-derived embryos. In the following sections, novel manuscripts that emphasize the roles of EVs from male and female reproductive fluids on reproductive processes are summarized. These insights on gametes and embryo production and culture must be considered to enhance the poor success rates of some of the ART procedures. For instance, an effective method for horse IVF [12] or a method to improve the less than 45 % of IVF/ICSI blastocyst rate in the ovine model [13] have not been established yet, despite decades of research.

## 2. Extracellular Vesicles (EVs) and Their Role on ART Outcome Improvement

Nowadays, neither the farm animal industry nor pet reproductive management can be conceived without the use of ART. However, improvement of IVF and embryo culture media are required to overcome differences in quality and developmental potential between in vivo- and in vitro-derived embryos. EVs, present in both the female and male reproductive tracts, play important roles in gamete maturation and ultimately in the fertilization process. The omission of EVs in current media used for ART is one of the causes that might explain the lower embryo quality obtained in these in vitro conditions.

EVs are defined as spherical bilayers containing proteins, genetic material and lipids that transport their cellular content (cargo) to other cells acting as intercellular communicators [14]. EVs can be classified according to the organ where are originated, in particular, for the reproductive tracts EVs are called: epididymosomes if derived from the epididymis; prostasomes if derived from the prostate; vaginosomes if derived from the vagina; uterosomes if derived from the uterus; and oviductosomes if derived from the oviduct. In addition, EVs can be classified according to their diameter size in microvesicles or ectosomes (100–1000 nm) or exosomes (30–100 nm) [15]. To avoid confusion, in this manuscript we refer to EVs based on their origin without considering the diameter size classification (see Table 1 for more classification details). In this section we focus on the role of EVs on the gain of gametes function as well as embryo development competence with special emphasis in those manuscripts whose findings are applied to the improvement of ART outcome.

### 2.1. Relationship between Spermatozoa and EVs as a Tool to Enhance ART Results

The spermatozoon is an extraordinary cell that is designed to survive in different microenvironments including a body where it was not created with the ultimate goal of fertilization. Due to the large amount of protamines that replace histones, sperm chromatin is highly compacted [16]. Thus, sperm are not able to transcribe gene information, do not synthesize proteins, and therefore, regulate their function by post-translational modifications [17]. Nevertheless, sperm are surrounded by a plasma membrane; and during their maturation in the epididymis, the ejaculation process, and along their journey through the female reproductive tract, sperm can acquire information from the surrounding milieu by exchanging information with EVs found in these fluctuating environments [18]. It has been shown that EVs cargos are up taken by sperm by a fusogenic mechanism of their membranes [19,20] or by lipid-rafts domain mediated-endocytosis [21].

Sperm are produced in the testis and transported to the epididymis. Sperm maturation occurs while the sperm transit from the caput region of the epididymis towards the cauda, where they are ultimately stored until ejaculation takes place [22]. Several works described the role of epididymosomes on the sperm maturation process through the transfer of cargo proteins and small RNAs to sperm (for review see [23]) while others focused on their applied roles on ART. Here we focus on the latter (Summarized in Table 2).

It has been shown in cats that epididymosomes affect sperm motility in vitro. Co-incubation of immature sperm obtained from the caput epididymis with epididymosomes for a short period of time (up to 1 h), showed a modest enhancement of total motility. Interestingly, longer exposure to epididymosomes (1.5 to 3 h) increased the percentage of sperm displaying progressive motility [33]. This could help maximize the chances of obtaining sperm with fertilization potential from epididymis of animals with high genetic value that present ejaculation issues or sudden deaths of endangered species.

At the moment of ejaculation, sperm get exposed to EVs-containing seminal fluid originated in three accessory glands: the seminiferous vesicle, the bulbourethral glands, and the prostate. Nevertheless, prostasomes are the most widely studied EVs in the seminal fluid. Once ejaculated, sperm initiate their journey through the female reproductive tract. The acquisition of hyperactivated motility, the ability to undergo the acrosome reaction, and, at the molecular level, the increase of protein tyrosine phosphorylation have been historically used as hallmarks of capacitation status [37,38].

Contradictory results have been found on the role of prostasomes on sperm capacitation. On one hand, a protective function against premature capacitation and acrosome reaction has been described on human and stallion sperm [24,25,39,40]. These findings were associated to the membrane composition of EVs with high content of cholesterol and sphingomyelin that decrease fluidity of sperm plasma membrane once the EVs and sperm fusion occurs [40,41]. The inhibition of premature capacitation might have a functional application on ART. For instance, the prevention of premature sperm capacitation is desirable when seminal doses are stored before performing AI or to counteract capacitation-like events during the sperm cryopreservation procedure [42]. In addition, an exhaustive work described that prostasomes added to boar seminal doses preserved at 17 °C for a long-term period was able to: (1) prolong sperm motility; (2) increase the total sperm antioxidant capacity, and; (3) protect plasma membrane integrity [31]. In that work, they also showed that prostasomes protection of sperm against premature capacitation was associated to their seminal plasma protein 1 (PSP-1) and carbohydrate-binding protein AWN (AWN) cargo. Nevertheless, prostasomes did not affect sperm ability to undergo capacitation when they were stimulated [31]. On the other hand, other authors described that in vitro incubation of boar sperm with isolated prostasomes enhanced the acrosome reaction [30]. Interestingly, qualitative but not quantitative differences were found on prostasomes of normozoospermic and severe asthenozoospermic men [43]. Prostasomes from normozoospermic men transferred cysteine-rich secretory protein 1 (CRISP1) to sperm [43]. CRISP1 is a protein with the ability to regulate murine CatSper channel enhancing the acrosome reaction induced by Ca^2+^ ionophore [27] and also to participate in the sperm-zona pellucida binding through the interaction with ZP3 [44]. Similarly, the plasma membrane calcium ATPase pump 4 (PMCA4), a vital machinery to regulate sperm calcium homeostasis was delivered in vitro into spermatozoa either by epididymosomes [45] or by prostasomes [46]. Future research on prostasomes must explore their applied function on ART considering that variation between species might be found that could be related to differences in their evolutive reproductive strategies.

In most species, sperm are deposited in the vagina during ejaculation and spend there a short period of time before continuing to travel through the female reproductive tract. This short time in the vagina is sufficient for sperm to be exposed to vaginosomes (VGS), which could have an effect on sperm functionality. Mice sperm incubated in non-capacitating conditions exposed for 30 min to VGS presented an enhanced progesterone-induced acrosome reaction [29]. In addition, co-incubation of sperm with vaginal luminal fluid (containing VGS) resulted in sperm incorporation of SPAM1, PMCA1/4, PMCA4, all proteins with roles on calcium homeostasis and the capacitation process as well as an overall increase in sperm protein tyrosine phosphorylation [29]. The transfer of tyrosine phosphorylated proteins by VGS could explain why sperm lacking the tyrosine kinase FER do not display tyrosine phosphorylation and do not fertilize in vitro although they are able to fertilize in vivo [47].

After leaving the vagina sperm enter the uterus. In vitro studies have shown that a short exposure (15 min) of sperm with uterosomes secreted by endometrial epithelial cells simulating the time that they spend in the uterus is enough to enhance sperm capacitation status in human spermatozoa [26]. Others authors have described the same results but with longer exposure times [21].

Finally, sperm pass through the uterotubal junction and reach the oviduct where the encounter with the oocyte and fertilization take place. Here, sperm interact with oviductosomes (OVS) produced by the oviductal epithelium. The impact of OVS on sperm function has been studied in several species. In mice, the OVS cargo is incorporated into sperm and is responsible for the rise of the protein PMCA1 and an increase in tyrosine-phosphorylated proteins levels in sperm [48]. Interestingly, it was observed that capacitated spermatozoa uptake higher quantity of OVS cargo that their non-capacitated counterparts [48]. These results were associated to a higher plasma membrane permeability found in capacitated spermatozoa since they lost sterol during the capacitation process [48].

In the bovine model, OVS have been used as a supplement of non-capacitating media for thawing cryopreserved sperm. The results obtained were dependent on the OVS origin: ampulla or isthmus. After incubation with isthmus-originated OVS, sperm displayed characteristic features associated to control capacitated sperm such as high levels of protein tyrosine phosphorylation, increased acrosome reaction and intracellular calcium responsiveness to progesterone [36]. The co-incubation of sperm with ampulla-originated OVS displayed an augmented capacitation response even when compared to capacitated control [36]. In summary, incubation of non-capacitated spermatozoa with OVS induced sperm capacitation and enhanced sperm survival with similar levels to those described in capacitated spermatozoa [36].

The role of OVS on sperm function was also studied in cats. In vitro experiments showed that cat’s OVS bind to the acrosomal region of the sperm head and to the mid-piece of the sperm tail. In addition, OVS used as additive to regular capacitating media enhanced the percentage of motile sperm as well as increased the rates of cleavage and blastocyst formation (23% and 8%, respectively) in comparison with control (no OVS co-incubated) [34]. The authors then investigated the cargo proteins in the OVS by mass spectrometry. The analysis of protein content of OVS identified a total of 4879 proteins, and between other functions, proteins involved on the sperm-oocyte interaction and fertilization process as cluster differentiation 9 (CD9), CCTs (cytosolic chaperonin containing TCP-1;) and TCP1 were found [34].

It has been shown in mice that EVs derived from either the epididymis or the oviduct can transfer miRNAs into the sperm [49,50]. For instance, OVS miR-34c-5p is delivered to the sperm [50] and has an important role on the fertilization process by initiating the first cleavage division. Bypassing the interaction between sperm and OVS as occurs in in vitro conditions could lead to the failure of first cleavage division. Consequently, it might negatively impact ART results where for example a single semen donor can be used to inseminate hundreds of females.

Besides the studies focusing on EVs obtained from the reproductive tract, other study evaluated the effect of EVs obtained from adipose-derived mesenchymal stem cells (ASCs) cultivated in vitro and used as additive to dog sperm cryopreservation media [35]. Surprisingly, ASCs-EV lead to a significant improvement of sperm motility and mucus penetration ability [35]. In addition, ASCs-EV protected spermatozoa against damage of the plasma membrane, the acrosome membrane, and the chromatin. The authors associated EVs beneficial effects during the freezing/thawing process to their protein and mRNA cargo associated to plasma membrane and chromatin repair process [35].

**Figure 1:**
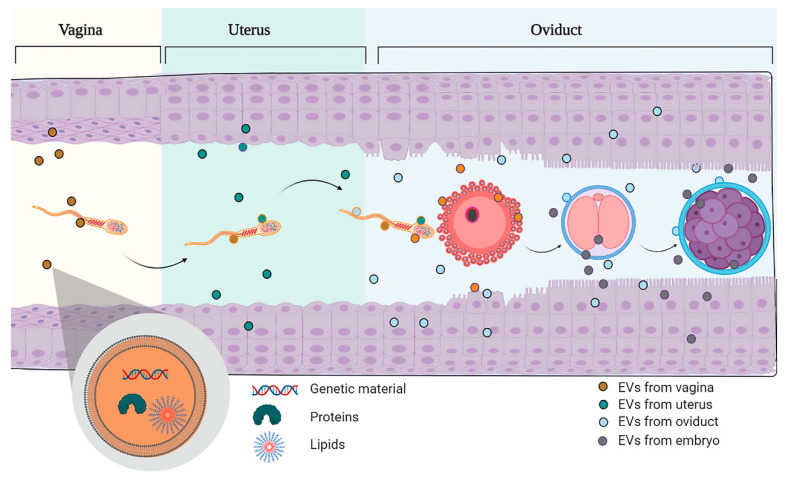
Schematic representation of the sperm travel through the female reproductive tract. Sperm enter in contact with the different extracellular vesicles produced in the vagina, the uterus and the oviduct. Once fertilization takes place, the embryo will come into contact with the EVs produced by the oviduct and the uterus where the embryo and the future fetus will remain for the rest of the pregnancy until delivery. Note that the embryo also produces EVs that allow bidirectional communication with the mother tissue (oviduct).

In summary, sperm receive pivotal elements through their interaction with EVs produced in the different sections of the male and female reproductive tracts (Figure 1), and these evidences should not be underestimated for the development of future enhanced sperm culture media that mimic physiological environmental conditions.

### 2.2. Relationship between Oocyte Maturation and EVs Used as a Tool to Enhance ART Results

In most mammalian species, before ovulation, oocytes are in an immature stage (germinal vesicle (GV)), in order to be competent for fertilization, they need to undergo meiotic resumption and arrive to the meiotic competence stage (metaphase II (MII)). Collection of immature GV oocytes from the ovaries followed by incubation in specific conditions that allow for the resumption of meiosis is a common practice in ART such as IVF [51]. This process is named in vitro maturation (IVM) and current IVM media described are deficient to obtain a proper oocyte maturation in some species [52,53]. For instance, the canine industry also has to address the issue of low efficiency of oocytes IVM. Thus, OVS used as additive along the canine oocyte IVM renders better results in comparison with control 21.82% and 8.66%, respectively [52]. Recently, it has been elegantly demonstrated that the percentage of mature canine oocytes after IVM in presence of OVS is enhanced through OVS cargo [54,55]. Leet et al., demonstrated that EGFR/MAPK signaling is the responsible for this improvement by the use of an inhibitor of this pathway (gefitinib) and an inhibitor of exosomes generation (GW4869) [54]. In addition, canine OVS enhance antioxidant capacity, viability and proliferation of canine cumulus cells [54].

In the ovary, oocytes are contained in follicles that will gradually mature from primordial into pre-ovulatory. The latter are follicles larger in size, filled with follicular fluid, and composed by theca and granulosa cells that surround an oocyte. It has been shown in several species that the follicular fluid contains EVs and that these EVs may have a role in cellular communication within the follicle [56,57]. In bovines, EVs from follicular fluid induce granulose cells proliferation through Src, PI3K/Akt and MAPK signaling pathways [58]. In addition, granulose cells preferentially uptake EVs from small over EVs from larger follicles [58]. The follicular fluid of mares contains EVs, and their proteins and miRNAs cargo were analyzed and described as a pathway of communication between oocytes and ovaries [56]. It was elegantly shown—in both in vivo and in vitro conditions—that EVs from follicular fluid are uptaken by the granulosa cells that surround the oocyte [56]. Interestingly, the authors described that miRNAs’ EVs cargo from follicular fluid changes along with the age of the mare and this fact might explain age-related decline of oocyte quality in this species [56]. Interestingly, EVs isolated from ovarian follicular fluid used as an additive during bovine oocyte maturation and embryo development in in vitro conditions enhanced blastocyst rate and decreased global DNA methylation and hydroxymethylation levels [59]. Nevertheless, caution needs to be taken when using follicular fluid EVs as additive to enhance oocyte competence, as it was shown that the EVs cargo vary along the estrus cycle [60,61,62], the size of the follicle [63], and with the age of the female [56].

Another clear example of EVs used to enhance current ART was shown when EVs isolated from follicular fluid were added during the process of vitrification/thawing of immature cat oocytes, a procedure that compromises oocytes ability to undergo meiotic resumption [64]. In this case, the addition of EVs did not protect against the loss of oocyte viability during vitrification/thawing but enhanced the oocyte IVM rate after vitrification as higher numbers of oocytes arrived to MII stage (28.3%) in comparison with controls (8.6%) [64].

### 2.3. Relationship between EVs Used as a Tool to Enhance Embryos and Conceptus Development Obtained by ART

After ovulation, oocytes transit through the oviduct from the ampulla towards the isthmus. Fertilization occurs in the ampulla and is followed by the initiation of embryo development. Early embryo development and the transit of the embryo towards the uterus occur simultaneously. Valuable information was acquired in the 90s when it was described that embryos cultivated in groups displayed better cleavage and blastocyst formation rates than those incubated individually [65]. This fact has been associated to embryo secretion of autocrine/paracrine growth factors (secretome) that lead to a better embryo development [66,67]. In addition to the secretome, it was confirmed that EVs produced by the embryos contribute to the better embryo competence when they are cultured together [68]. Hence, information carried by embryo-derived EVs is not only important to communicate with the female tract, but also to communicate between them and achieve better embryo competence.

The oviduct has an important role in fertilization and embryo development. It is not surprising to find that the oviductal fluid of several species contains EVs [19,34,48,69,70], and as mentioned above these EVs were specifically named oviductosomes (OVS). The OVS cargo varies along the estrus cycle [48,71]. For example, OVS cargo of plasma membrane Ca^2+^-ATPase (PMCA), with Ca^2+^ clearance-homeostasis role [72], changes along the estrus cycle where the PMCA levels in proestrus/estrus are higher than in metestrus/diestrus [48]. Interestingly, it was shown that the concentration and size of OVS is stable along the bovine estrus cycle; however the OVS content varies [71]. For example, higher mRNA composition was found in OVS recovered in the post-ovulatory stages when compared to OVS recovered during the rest of the cycle [71]. Differences were also found at the protein level, and the major differences were found between OVS recovered at the post-ovulatory and pre-ovulatory stages [71]. Similarly, differences were found along the estrus cycle when the protein cargo of porcine OVS was analyzed [62]. Due to the variations found during the estrous cycle, the authors hypothesized that OVS cargo changes are regulated by hormonal changes during the estrous cycle [62,71].

Primary cultures of bovine oviductal epithelial cells (BOECs) in monolayers are commonly used as an in vitro model for the study of gametes/embryo interaction with the oviduct in the bovine model [73,74]. It has been shown that BOECs secrete EVs [75]. The supplementation of the embryo culture media with BOEC-derived EVs did not affect embryo development outcome. However, these BOEC-derived EVs improved cryotolerance of embryos vitrified as they increased survival rate and number of cells, and upregulated genes related to implantation (PAG1) and metabolism (GADPH) [75]. In that work, the authors also described a negative effect of EVs present in fetal calf serum (FCS), a common component used on embryo culture media, over the bovine embryo vitrification/thawing process [75]. Oviductal region specificity was evidenced as isthmus-derived EVs were more effective than ampulla-derived EVs [76]. Interestingly, similar results were found when OVS were used to supplement in vitro bovine embryo cultures: the OVS did not have any effect on embryo development rate but improve embryo cryotolerance [76]. Almiñana and collaborators showed that the addition of oviduct-derived EVs to the culture media did not improve IVF fertilization rates; although, it enhanced the blastocyst embryo quality and the embryo hatching rate [70]. They also showed that frozen EVs had better reproductive outcome (as hatching rate) when used as additive to the embryo culture media in comparison to fresh EVs [70]. These results highlight that EVs can be frozen without any detriment in their cargo capacities simplifying the logistics of their application to different ART.

**Figure 2:**
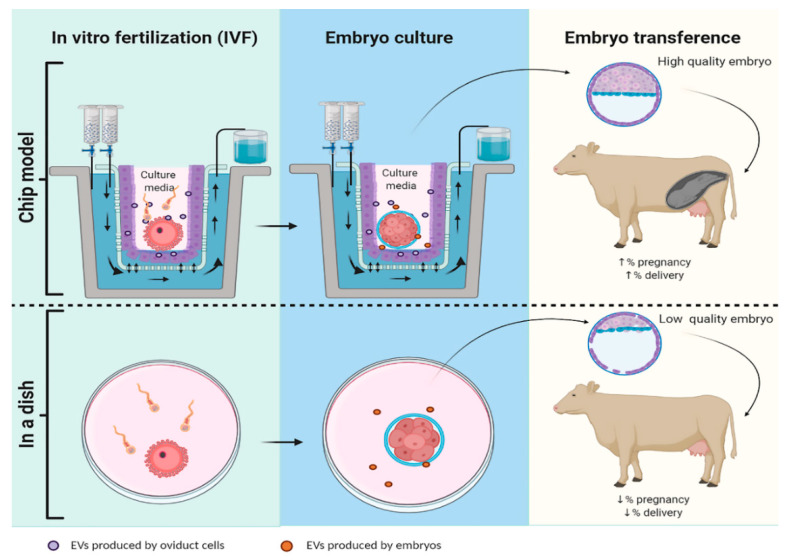
Schematic representation of embryo production using a Chip-model or an in vitro model (in a dish). Hypothetically in a Chip-model, the system mimics estrus cycle hormones concentrations that allows oviductal cells to develop cilia and produce EVs that will vary their cargo along the estrus cycle, increasing the pregnancy and delivery rate. In contrast, in embryo production by classic ART in a dish there is no interaction with EVs oviduct, consequently the embryos produced have lower quality and less chance of implantation and ending in delivery.

Despite similar results found by addition of BOECs-derived or oviduct-derived EVs to enhance ART outcome, it was revealed that there are qualitative and quantitative differences in the protein cargo between them [70]. A total of 319 proteins (47 only expressed in BOEC derived and 97 only expressed from oviduct derived) were identified by mass spectrometry, where only 175 of them were common to both populations [70]. Oviduct-specific glycoprotein 1 (OVGP1), a protein that enhances embryo development [77] and quality [78] was only present in in vivo EVs. One explanation for this discrepancy between in vitro and in vivo could be that static BOEC cultures induce cell dedifferentiation and therefore these cultured cells might lose some functions. Then, invaluable information could be obtained when using the novel dynamic culture system oviduct-in-a-chip developed by Ferraz an collaborators (2018). This culture system of oviductal epithelial cells allows the investigators to simulate physiological conditions by applying hormonal waves, and has shown to maintain epithelial cell differentiation and presence of cilia [79]. Interestingly, OVGP1 levels also increased when 3D-Chip were used to grow BOECs [79]. More cell culture studies using the chip strategy could help extrapolate results to in vivo conditions (see Figure 2). 

The use of OVS in ART was proven beneficial also in other species. In rabbits, embryos co-incubated with OVS decreased reactive oxygen species (ROS) and DNA methylation levels that lead to an increase of the blastocyst development rate [80]. The authors showed that the antioxidant properties were associated to the melatonin OVS cargo by the use of luzindole, a selective melatonin receptor antagonist, that attenuated the positive effect of OVS on embryos [80]. In pigs, OVS have been used successfully to face the problem of polyspermy in porcine IVF, doubling the percentage of oocyte penetrated by a single spermatozoon [69].

Another study using a murine model described that OVS obtained from pregnant females used as additive for the IVF procedure enhanced embryo transference efficiency in comparison with the supplementation with OVS obtained from pseudo-pregnant females [81]. Pregnant females OVS increased the percentage of blastocysts, and the embryo quality determined by an increase of gene expression related to successful embryo development (Bcl-2 and Oct-4), an increased inner cell mass: trophectoderm ratio, and a decreased embryo apoptosis. In addition, birth rates after embryo transference were also enhanced [81].

An appealing application of EVs for human ART was found by the use of EVs obtained from human endometrial-derived mesenchymal stem cells (EV-endMSCs) isolated from menstrual blood [82,83,84]. IVF-embryos obtained from aged murine females and supplemented with human EV-endMSCs in the embryo culture media presented enhanced embryo development. Furthermore, blastocyst rate was doubled by the addition of 20 µg/mL of EV-endMSC in comparison to controls [83,84]. Positive effects of EV-endMSC in the embryo competence and quality of those embryos obtained in aged oocyte were associated to different levels of mRNA expression of genes associated to cellular response to oxidative stress (Sod1), metabolism (Gadph), placentation (Vegfa) and trophectoderm/ICM formation (Sox2) [83]. Moreover, human EV-endMSC used as additive to the culture media of mouse zygotes obtained in vivo increased the number of total cells by blastocyst and the hatching rate [82]. Recently, EVs derived from human umbilical cord mesenchymal stem cells (HUCMSCs) were successfully used to restore fertility on female mice with premature ovarian insufficiency (POI) [85]. Isolated EVs-HUCMSCs administered by only one intravenous injection rescued ovarian function, hormones levels (FSH and E2), as well as enhanced the mating behavior and the numbers of pups born after four weeks of treatment [85]. In addition, the POI group treated with EVs-HUCMSCs increased parameters associated to IVF procedures such as number of oocytes retrieved, percentage of fertilized oocytes, cleaved embryos, and blastocyst rates although did not reach values of the control group [85]. The effects of EVs on ART and embryo development are summarized in Table 3.

Once fertilization has occured, the embryo starts a series of developmental changes that orchestrate the transformation from a zygote into a blastocyst. During this period of early embryonic development, the embryo is still enclosed in the zona pellucida (ZP, a glycoproteic layer that surrounds the plasma membrane of the oocyte). Embryos in blastocyst stage will hatch from the ZP and implant by attaching and invading the uterus of the mother. Thus, embryo-uterus interaction plays vital roles in the recognition of pregnancy, maintenance of the corpus luteum, and consequently the allowing for the progression of pregnancy [86]. It has been shown that exist differences between non and pregnates mares’ miRNA EVs cargo obtained from serum [87]. In adddition this EVs cargo might contribute to the pathway of maternal recognition of the pregnancy [87]. In other work it was shown that murine EVs produced by endometrium that contained miRNAs involved in the implantation process were internalized by embryo through the trophectoderm and increased embryo adhesion levels [88].

On the other hand, it was shown that conceptus-derived EVs also promote communication between the conceptus and the maternal tissue during the establishment of pregnancy in ovines [89]. In that study, the conceptus (14 days) originated in vivo were cultivated for a day in a dish and then the EVs were collected from the medium. Later on, the EVs originated by the conceptus in in vitro conditions were labelled (PKH67) and transferred to pregnant ewes. The authors showed that the embryos and the uterine epithelium of the pregnant female receptors internalized the conceptus-derived EVs [89]. In the same work, it was also shown that EVs obtained from the uterine luminal fluid are internalized by the conceptus trophectoderm and uterine epithelia [89]. This work ilustrates that embryo-derived EVs might have an impact on the receptivity of the uterus and that maternal-fetal communication could be key for a successful implantation and progression of pregnancy. Considering this, implantation failure of certain ART such as embryo transference might be related to the lack of embryo-mother communication during the first stages of development.

## 3. Challenges for the New Era of ART Development

Based on the broad-spectrum possibilities for EVs application, EVs have been postulated as potential non-invasive biomarkers. For example, EVs can be used as a biomarker for embryo quality [90,91,92] to select good embryos before transferring them to female recipients or to know the receptivity/health of a uterus before performing an embryo transference [93]. In addition, the increasing knowledge about the role of EVs on reproductive events and ART efficiency will lead to the next challenge for the biology of reproduction field: the large-scale production of EVs to use them as additives to gametes and embryo culture media. This might be addressed easily in livestock animals by the use of animal’s remains from abattoirs; where moreover this strategy is in accordance with the three rules of animal welfare. Similarly, EVs can be obtained from the biological material originated in the daily practice of vet clinics, as it is the castration of domestic animals (i.e., cats and dogs). Nevertheless, both strategies are time consuming and it might present contaminations with other biological fluids. The future of EVs development as a real alternative tool to boost ART results involves the optimization of the oviduct-in-a-chip model prototype to culture in vitro embryos before transferring them to female recipients (See Figure 2).

Another possible therapeutic application of EVs for ART is to use it as cargo of information that the gametes or embryos are lacking in current ART protocols to succeed in fertilization and embryo development. It has been shown that EVs can be loaded with the desired information by electroporation [94]. Then, EVs could be preloaded with the biological deficient information found for instance in proteins of sperm of asthenozoospermic men or with those miRNAs transported by sperm that have shown to enhance embryo development.

The study of EVs for ART should also contemplate possible adverse effects of EVs on specific ART outcome. EVs overdose presented detrimental effects over embryo development due to elevated levels of ammonium that might be correlated to the EVs protein cargo in mice [81]. Negative effects of OVS overdose were also found when these EVs were used for canine oocyte IVM (less oocytes arrived to MII phase), and this effect was associated to higher levels of miR-375 [52], a microRNA that has been linked to poor oocyte quality in aged mares [56]. Consequently, EVs titration obtained from every subsection of the reproductive tract must be performed, and their effects on gametes and embryos must be determined before the universalization of the use of EVs protocols. In addition, these protocols must be adapted to the specific species under study.

## 4. Concluding Remarks

Although this review focused on EVs used to enhance ART outcome in mammals, it should be noted that EVs have also drawn the attention of the avian reproduction sector as it has been recently postulated that uterosomes might have a role on avian sperm survival [95]. All the information described above should be considered for the development of improved media such as gamete collection, IVF, and embryo culture media used in vitro for ART. Besides the supplementation of media with specific EVs, the possibility of re-use EVs should be explored. For instance, once an in vitro embryo culture is finalized, the EVs contained in the media could be recovered, isolated and used as additive for a new culture media in those females with a record of low oocytes and embryo quality.

We expect that researchers working in the reproductive field will increase their interest in the study and use of EVs. This is a fundamental step for the development of new tools that could improve ART outcomes. From our point of view, the broad applications of EVs will have a lasting impact on the field of reproductive biotechnology.

## Figures and Tables

**Table 1:** Classification of extracellular vesicles (EVs) according to their localization in the reproductive tract or their diameter size.

Subsection	Name
Epididymis	Epididymosome
Prostate	Prostasome
Vagina	Vaginosome
Uterus	Uterosome
Oviduct	Oviductosome
**Size (nm)**	**Name**
100–1000	Microvesicles or ectosomes
30–100	Exosomes

**Table 2:** Effects of co-incubation of extracellular vesicles and spermatozoa on ART outcome.

Specie	EVs Origen	ART used	Output	Reference
Human	Prostate	In vitro incubation in acidic media	↑ % of motile spermatozoa	[24]
Human	Prostate	In vitro capacitation	Inhibit sperm capacitation Inhibit spontaneous acrosome reaction	[25]
Human	EECs	In vitro capacitation	Enhance sperm capacitation status	[26]
Human	Prostate	In vitro capacitation	Enhance acrosome reaction response to calcium ionophore	[27]
Human/ mouse	Prostate	In vitro incubation	↑ Hypermotility ↑ IVF fertility	[28]
Mouse	Vagina from superovulated females	In vitro capacitation	Enhance sperm responsiveness to progesterone Incorporation of several sperm proteins with roles on calcium homeostasis (SPAM1, PMCA1/4, PMCA4) and capacitation process (protein tyrosine phosphorylation)	[29]
Pig	Prostate	In vitro incubation	Enhance sperm acrosome reaction	[30]
Pig	Prostate	Preservation at low temperature	Prolonged sperm motility ↑ Sperm antioxidative capacity ↓ Lipid peroxidation Protect plasma membrane Protect against premature capacitation	[31]
Stallion	Prostate	In vitro capacitation	Inhibit sperm capacitation events as protein tyrosine phosphorylation	[32]
Feline	Epididymis	In vitro incubation	↑ % of motile spermatozoa for a short period of time (up to 1 h) ↑ Forward motility (1.5 to 3 h of co-incubation)	[33]
Feline	Oviduct (different follicular phases)	IVF	↑ % Motile spermatozoa Protect again premature acrosome reaction Enhanced IVF outcome	[34]
Dog	ASCs	Cryopreservation	↑ Sperm motility and viability ↑ Mucus penetration ability or ↓ Acrosome and chromatin damaged	[35]
Bovine	Oviduct (different sections)	Cryopreservation	↑Protein tyrosine phosphorylation ↑ Responsiveness to progesterone Maintain sperm survival	[36]

List of abbreviations: EECs: endometrial epithelial cells; ASCs: adipose-derived mesenchymal stem cells; SPAM1: sperm adhesion molecule 1; PMCA: plasma membrane calcium-transporting ATPase; ↑: increase; ↓: decrease.

**Table 3:** Effects of co-incubation of extracellular vesicles and embryo on ART outcome.

Specie	EVs Origen	ART Used	Output	Reference
Bovine	BOEC	IVC Vitrification	No differences on embryo development Enhance vitrification outcome: ↑ Embryo quality ↑ Cryo-survival rate ↑ Number of cells	[75]
Bovine	BOEC	IVP IVC	Enhanced the embryo quality ↑ Number of cells ↑ Hatching rate = Fertilization rate	[70]
Bovine	Oviduct (ampulla and isthmus)	IVC Vitrification	No differences on embryo development ↑ Cryo-survival rate	[76]
Mouse	endMSCs	Embryo culture obtained in vivo	↑ Number of total cells by blastocyst ↑ Hatching rate	[82]
Mouse (ageing)	endMSCs	IVF IVC	Enhance embryo competence and quality ×2 blastocyst rate ↑ mRNA expression of Sod1, Gadph, Vegfa and Sox2	[83,84]
Mouse	Oviduct from pregnant females	IVF ET	↑ Embryo quality (↑ Bcl-2; Oct-4↓Bax) ↑ ICM ↑ Blastocyst and birth rates	[81]
Mouse (POI)	HUCMSCs	IVF	Rescue ovary function, hormones levels (FSH and E2), natural fertility. ↑ oocytes retrieved, fertilized zygotes, cleaved embryos and blastocysts	[85]
Porcine	Oviduct	IVF	↓ Polyspermia	[69]
Feline	Ovarian fluid	Vitrification IVM	= Vitrification survival rate ↑ Oocyte IVM from 8.6% in control to 28.3% in supplemented with EVs	[64]
Canine	Oviduct	IVM	↑ Oocyte IVM 21.82% vs. control 8.66%	[52]
Canine	Oviduct	IVM	↑ Cumulus cell viability, and proliferation rate ↓ ROS and apoptotic rate	[54]
Canine	Oviduct	IVM	↑ Maturation rate of oocytes	[55]
Rabbit	Oviduct	IVF IVC	↓ ROS and DNA methylation levels ↑ Blastocyst rate	[80]

List of abbreviation: Bax: BCL2-associated X protein; BOEC: bovine oviduct epithelial cells; E2: estradiol; ET: embryo transference; endMSCs: human endometrial-derived Mesenchymal Stem Cell; FSH: follicle stimulating hormone; Gadph: glyceraldehyde-3-phosphate dehydrogenase; HUCMSCs: human umbilical cord mesenchymal stem cells; ICM: inner cell mass; IVC: in vitro culture; IVM: in vitro maturation; Oct-4: transcription factor-1; POI: premature ovarian insufficiency; ROS: reactive oxygen species; Sod1: superoxide dismutase 1; Sox2: SRY-related HMG-box gene 2; Vegfa: vascular endothelial growth factor A. ↑: increase; ↓: decrease.
